# Veterinary Department, Harvard University

**Published:** 1890-07

**Authors:** 


### VETERINARY COLLEGE NOTES.

*Veterinary Department, Harvard University.*—At the commencement exercises of the Veterinary Department, Harvard University, held at Saunders' Theatre, Cambridge, Mass., on June 25th, the President conferred the degree of M.D.V. upon the following gentlemen, six in number: Edward Thomas Harrington, Joseph Michael Kiggen, William Safford Lord, Edward Patrick McKenna, Wilbert Soule, Frederick Hiram Gage.

### ETYMOLOGY OF THE WORD "VETERINARY."

In his magnificent work, *De Verborum Significatione*, of which we, unfortunately, know but a part in its original form, the grammarian, Ferrerius Flaccus (died 14 A.D.), comprehends in the common denomination of *Bestia veterina* all those animals which work under the yoke, and derives this word from *veho* (I draw). From that, the *veterinarius* would be the man who occupies himself with animals of draught, in whatever manner it may be. Cato is of the same opinion. Opilius thinks that the word comes from the fact that the animal, designated by *veterina animalis*, bears its load attached to the belly *ad ventrem onus religatum*, and that it should be called *venterina*, not *veterina*. Varro

(116 A.D.), is of this opinion in comprehending by *ceteræ veterinæ*, the "other pack animals."

The opinion of Columella is the most plausible ; he derives *veterinarius* from *vetus* (aged), because the shepherds, the most aged, instructed their young companions in the treatment of farm animals. He says : *Quarre veterinariæ medicinæ prudens esse debit pecoris magister.*

Hensinger makes the word come from the Sanscrit by successive derivation of *pæn*, *pecus*, *faihus*, *feh*, which is decidedly too far fetched.—*Monatschrift d. Ver d. Thierärzle in Œsterreich.*

